# COVID-19 Pandemic Impact on Essential Public Health Services and Online Health Communication—Appalachian Kentucky, March–December 2020

**DOI:** 10.13023/jah.0402.03

**Published:** 2022-07-01

**Authors:** Margaret A. Riggs, Kenny Ortiz-Jurado, Keith Klein

**Affiliations:** Centers for Disease Control and Prevention, margo.riggs1@gmail.com; Shaping Our Appalachian Region; Wabash College

**Keywords:** Appalachia, COVID-19, Public Health Services, Rural Health, Health Communication, Telehealth

## Abstract

**Introduction:**

The COVID-19 pandemic posed many challenges for local health departments (LHDs). This study examines how stay-at-home orders impacted the provision of essential public health services and subsequent increased use of online health communication by LHDs for rural populations in Appalachian Kentucky during the first year of the COVID-19 pandemic.

**Methods:**

A survey to gather information about provision of essential public health services was administered to five LHDs representing 13 counties between June 2020 and December 2020. Additionally, demographic and health-outcome data from LHD, state health department, and CDC websites were reviewed, alongside health communications posted on LHD social media pages. Using these data, the authors conducted descriptive analyses to assess how essential public health services and health communications were impacted by the COVID-19 pandemic.

**Results:**

In Appalachian Kentucky, limited internet access was identified by all LHDs as the most common barrier for clients of essential public health services. During stay-at-home orders, the use of telehealth increased participation rates for programs that support parents for optimal growth and development of children. Additionally, social media was the most preferred media source by all LHDs to communicate with the local population to promote health education.

**Implications:**

By using publicly available data and conducting interviews with LHDs, alongside examination of the health information they posted online, the study is able to characterize the impacts of the COVID-19 pandemic on providing essential public health services—successes and challenges. Expanding use of telehealth for essential public health services and increased access to online health communication improves access to care and information for rural populations in Appalachian Kentucky.

## INTRODUCTION

**Figure 1:**
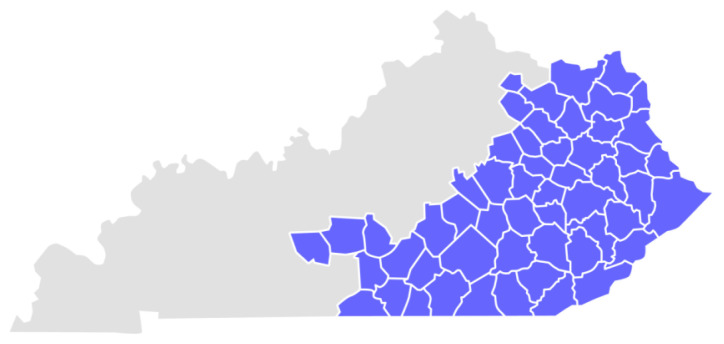
Appalachian Kentucky Counties Notes: Appalachian Kentucky counties are highlighted in purple. Map extracted from https://lek.eku.edu/insidelook/soar-regional-blueprint.

The mission of public health to improve health and achieve health equity has been challenged by the overwhelming demands of the COVID-19 pandemic.^1^ COVID-19 is caused by the severe acute respiratory syndrome coronavirus 2 (SARS-CoV-2) that is spread from person to person through respiratory droplets. This zoonotic virus was identified in Wuhan, China, in December 2019 and has since infected an estimated 534,000,000 worldwide, including over 6,000,000 deaths.^2^ With little to no pharmaceutical treatment available when the virus first emerged in late 2019, public health prevention strategies and implementation of non-pharmaceutical interventions were paramount for communities to navigate the pandemic successfully.^3^,^4^ Appalachian Kentucky consists of 54 counties with a total population of approximately 1,163,000 (Figure 1). Most counties are classified as economically distressed and experience health-outcome disparities.^5^ Specifically, rates of obesity, mortality rates from chronic obstructive pulmonary disease and heart disease, diabetes prevalence, and poverty rates have been historically higher in Appalachian Kentucky than in Kentucky or the U.S. as a whole.^6^,^7^ Moreover, Appalachian counties across the entire region in the U.S. have higher disparities in healthcare coverage, cost, and access to care than rates in their respective states and nationally.^6^,^7^ Understanding how public health organizations adapt their services and respond to pandemics such as COVID-19 may help communities better prepare for successful mitigation of disease outbreaks in the future.^8^

On March 6, 2020, Kentucky Governor Andy Beshear announced the Commonwealth’s first confirmed case of SARS-CoV-2 infection, and on March 13, 2020, the President of the United States declared a national emergency.^7^ Communication and public health education from local health departments (LHDs) to their communities had to be conducted on a daily basis to stay up to date with rapidly changing information. Social media was utilized more by LHDs than in the past. Disruptions in services provided by LHDs (and the wider healthcare system) were anticipated when, to reduce the spread of COVID-19, Kentucky limited movement outside homes only to essential activities through its “Healthy at Home” stay-at-home order on March 26, 2020.^9^ In the Commonwealth, LHDs provide essential public health services for their communities through a multitude of programs. Examples include Health Access Nurturing Development Services (HANDs); the Special Supplemental Nutrition Program for Women, Infants, and Children (WIC); the Sexually Transmitted Disease (STD) Prevention and Control Program/Family Planning, Immunization Program; the Harm Reduction Program; the Diabetes Prevention Program; the Tobacco Prevention and Cessation Program; and the Nutrition and Community Health Program (see Appendix A in Supplemental Files). Often, LHDs are the only source for these services within their communities.^10^ Communication from LHDs was imperative to prevent spread of SARS-CoV-2 and to inform communities of available essential services.

The Kentucky Healthy at Home order, while important for preventing further spread of COVID-19, posed additional challenges for a region struggling with access to adequate health care and suppression of local economies. LHD directors identified several challenges in particular:

- determining which essential services to continue and which services needed to be paused or referred to another clinic;
- ensuring sufficient allocation of supplies and resources needed for services to operate (including personal protective equipment);
- patient screening before entering facilities; and
- determining the impact of social media use in Appalachian Kentucky to share information during the COVID-19 pandemic.

Shaping Our Appalachian Region (SOAR) is an Appalachian Kentucky-based nonprofit that works to improve health in the region, encourage economic development, and reduce social vulnerability. Upon learning about the impact of the Healthy at Home order on limiting access to the essential public health services provided by LHDs, SOAR began urgent research to explore these effects of the pandemic.

## METHODS

### Data Collection Process

SOAR conducted this study from June 1, 2020, to December 31, 2020—at the request of LHDs—to assess both the impact of the Healthy at Home order on the provision of essential public health services and the impact of online health communication by LHDs (via social media) during the first year of the COVID-19 pandemic. The study used mixed methods that consisted of a survey tool, aggregating COVID-19 data for the Commonwealth and counties, and qualitative social media metrics (COVID-19 health education posts and responses) gathered from November 1, 2019, to December 31, 2020.

A standardized questionnaire was administered, via email or interview, to five LHD directors or their chosen representative. These five LHD directors volunteered to participate and represented 13 counties.

Existing aggregate COVID-19 data for the Commonwealth and its counties were extracted from the CDC^11^ and Kentucky Department for Public Health (KDPH)^12^ websites to identify the numbers of COVID-19 cases, incidence rates, positivity rates, and fatality rates. Additional demographic data (population, poverty rates) and health data (drug overdose deaths, diabetes, and obesity rates) were extracted from the Economic Research Service,^13^ Kentucky Health Facts,^14^ and Kentucky Drug Overdose Mortality Dashboard^15^ databases.

Social media data were collected from official Facebook accounts for the Breathitt County Health Department,^16^ Cumberland Valley District Health Department,^17^ Floyd County Health Department,^18^ Kentucky River District Health Department,^19^ and Pike County Health Department.^20^ Facebook posts and feedback responses received from the public were collected from November 2019 to December 2020 to explore how LHD used the social media platform to engage with the public. The activity was reviewed by CDC and conducted consistent with applicable federal laws and CDC policy.

### Sample

Three area development districts (ADDs) in rural Eastern Appalachian Kentucky (Big Sandy, Cumberland Valley, and Kentucky River) participated in this study. Kentucky is divided into 15 ADDs, which include all 120 of the state’s counties.

**Figure 2:**
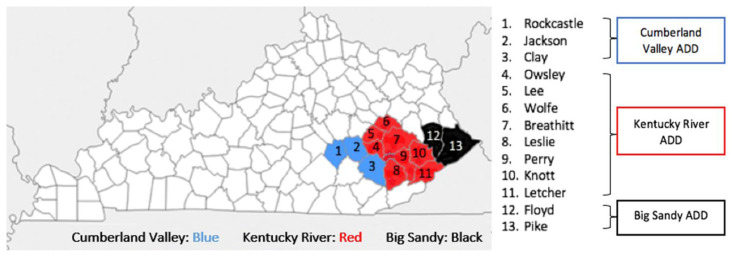
Participating Local Health Department Counties Notes: Cumberland Valley ADD counties are in blue (1–3); Kentucky River ADD counties are in red (4–11); and Big Sandy ADD counties are in black (12, 13).

The three ADDs in this study represent 13 counties: Big Sandy ADD (Floyd and Pike counties), Cumberland Valley ADD (Clay, Jackson, and Rockcastle counties), and Kentucky River ADD (Breathitt, Knott, Lee, Leslie, Letcher, Owsley, Perry, and Wolfe counties), as shown in Figure 2. The objective of ADDs is to foster regional collaboration by advancing the concept that local governments and business leaders can accomplish more goals by working together than by operating individually.

### Survey Tool

The survey consisted of 20 questions (multiple choice, open ended, and follow-up) grouped into three sections:

- **Section A** consisted of four items about each county: population demographics, obesity rate, drug overdose deaths, and poverty rates (see Appendix B in Supplemental Files).
- **Section B** consisted of three key metrics before and during the Healthy at Home order (see Appendix B in Supplemental Files):
  1. most frequently used LHD essential public health services;
  2. success identified as access to and utilization of telehealth services; and
  3. challenges faced due to lack of supplies or resources necessary for services to operate during the COVID-19 pandemic.
    Participants were asked how the COVID-19 pandemic impacted nine essential services available in Kentucky at LHDs: HANDS, WIC, STD Prevention/Family Planning, Immunization, Harm Reduction, Diabetes Prevention, Tobacco Prevention, and Cessation, Nutrition, and Community Health (see Appendix A in Supplemental Files for details).
- **Section C** gathered information about participants’ preferred forms of public health communication during COVID-19, including questions about social media content, public feedback, and the media platform considered most effective in promoting public health awareness during the Healthy at Home period (see Appendix C in Supplemental Files).

### Data Analysis

Descriptive statistics were calculated using Excel summary statistics for the following metrics: population demographics and health outcomes, COVID-19 impact on LHD essential services, successes with telehealth, challenges associated with lack of supply or resources to continue operations during the Healthy at Home order duration, LHD public health communication, and Facebook posts and feedback responses for evaluating online health communication (via social media) and public engagement.

To assess the success of telehealth, responses to the survey tool were tagged and classified into two main categories: *moderate increase* or *substantial increase*. The *moderate increase* category included comments such as “we have seen an increase in participation and compliance” and “program was successful.” The *substantial increase* category included comments such as “currently have the highest participation we have ever seen” and “there was a significant impact on participation.” To ensure inter-rater reliability after the first reviewer completed the rating, a second reviewer independently rated a subset of data randomly chosen for one month from each LHD: Cumberland Valley Department of Health (December 2019), Floyd County Health Department (June 2020), Pike County Health Department (September 2020), Breathitt County Health Department (July 2020), and Kentucky River District (October 2020).

Facebook posts made by the LHD on their official sites and feedback responses submitted by the public onto the posts were tracked manually by one reviewer and categorized independently by two reviewers who came to a consensus for each month of data to assess how often LHDs used the platform and what type of feedback response was received for each post. Feedback responses on Facebook posts were interpreted using three metrics: positive, negative, and neutral/inconclusive. Positive feedback included comments of gratitude or encouragement, such as “thank you for keeping us informed,” “please continue practicing social distancing and wearing a facial mask,” “we can do this together,” and the use of “like” and “love” emojis. Negative feedback included critical comments, such as “number of confirmed cases look [sic] suspicious,” “It is all a conspiracy,” “I will not wear a mask anywhere,” or the use of “angry” emojis. Lastly, feedback was determined to be neutral or inconclusive if it contained emojis and comments that did not elicit any positive or negative response or included general questions about the COVID-19 response, such as “when are new confirmed cases updated?” or “I heard that people contracted the virus at church, school” or “ok.” Inconclusive Facebook emoji reactions included the “laugh”, “sad”, and “surprised face” emojis.

## RESULTS

### Demographics and Health Outcomes

Cumberland Valley ADD consists of eight counties—Bell, Clay, Harlan, Jackson, Knox, Laurel, Rockcastle, and Whitley—with a total population of 238,521 (as of 2019), an average obesity prevalence of 20%, an average poverty prevalence of 27%, and 76 reported overdose deaths in 2019 (Table 1). Data for this study were collected from three of the eight counties that chose to participate: Clay, Jackson, and Rockcastle. Of these three counties, Clay County has the largest population (20,368), the highest poverty rate (33%), and had eight overdose deaths in 2019. However, Clay County had the lowest rates of diabetes (18%) and obesity (42%) (Table 1).

Kentucky River ADD consists of eight counties: Knott, Lee, Leslie, Letcher, Owsley, Perry, Wolfe, and Breathitt. Together, these counties have a total population of 105,992 (as of 2019); prevalence of obesity (45%), diabetes (18%), and poverty (31%); and 60 reported overdose deaths (shown in Table 1). Data were collected for the Kentucky River District Health Department (which serves Knott, Lee, Leslie, Letcher, Owsley, Perry, and Wolfe counties) and the Breathitt County Health Department. Of these eight participating counties, Perry County has the largest population (26,624) and one of the highest obesity rates (48%). Conversely, Perry County has the lowest prevalence of poverty (24%) and diabetes (15%) and had five overdose deaths in 2019 (Table 1).

Big Sandy ADD consists of five counties: Floyd, Pike, Johnson, Magoffin, and Martin. With a total population of 142,726 (as of 2019), its prevalence of obesity (44%), diabetes (24%), poverty (24%) and 61 overdose deaths are shown in Table 1. Data were collected from Floyd and Pike counties. Compared with Floyd County, Pike County has a larger population (59,497 versus 36,456), slightly higher diabetes prevalence (24% versus 23%), and more overdose deaths, with 23 occurring in 2019 (Table 1).

### COVID-19 Summary

**Figure 3:**
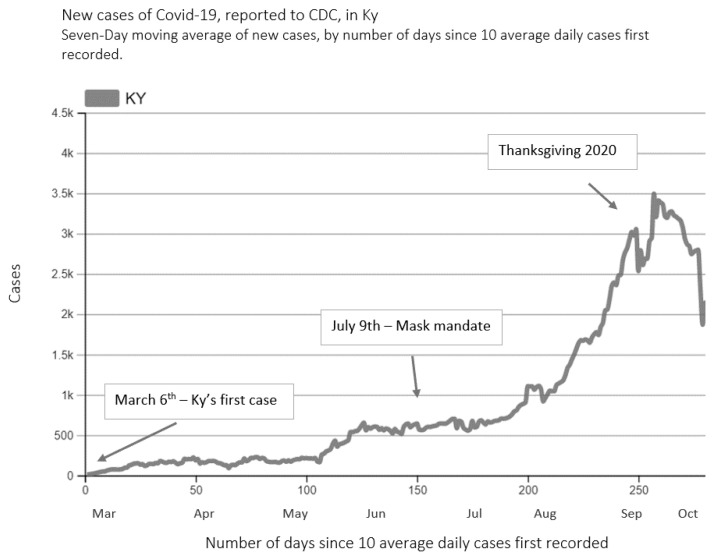
New COVID-19 Cases in Kentucky, March–December 2020

The first COVID-19 case was identified in Kentucky on March 6, 2020, and a total of 269,381 cases were identified in the Commonwealth by December 31, 2020.^7^ As Figure 3 demonstrates, following the identification of the first case, daily trends showed a slight increase in the number of cases over the summer, starting after Independence Day (July 4), which resulted in Kentucky’s governor instituting a mask mandate on July 9. There were two additional surges of COVID-19 cases in Kentucky that correspond to the Thanksgiving and Christmas/New Year holiday periods. By December 31, 2020, Kentucky was designated as *red* under CDC categorization (indicating substantial transmission with 100 or more cases per 100,000 population) and had the 35^th^-highest rate in the nation.^9^,^10^

At the same time, the Commonwealth was designated as *red* for test positivity (indicating a rate at or above 10%), with the 21^st^-highest rate in the country.^9^ From March 6 to December 31, 2020, Kentucky had 293 new cases per 100,000 population, compared to the national average of 391 per 100,000 population.^9^,^10^ According to LHD reports, the dates of the first confirmed cases were as follows: March 21, 2020 (Kentucky River ADD), March 23, 2020 (Big Sandy ADD), and March 31, 2020 (Cumberland Valley ADD).

As of December 31, 2020, Big Sandy ADD had identified a total of 7,331 cases, 76 deaths, and an incidence rate of 37 per 100,000 population. Cumberland Valley ADD had identified a total of 15,059 cases, 116 deaths, and an incidence rate of 69 per 100,000 population. Kentucky River ADD had identified a total of 6,129 cases, 69 deaths, and an incidence rate of 49 per 100,000 population (Table 2).

### Impact of COVID-19 on LHD Programs

#### Use of LHD Services Prior to Healthy at Home Order (before July 2020)

From the nine LHD programs (HANDs, WIC, STDs/Family Planning, Immunization, Harm Reduction, Nutrition, Community Health, Diabetes Prevention, Tobacco Prevention and Cessation), participants were asked to identify frequently used programs prior to the Healthy at Home order. HANDs and WIC (13/13) were frequently provided by all counties prior to Healthy at Home; Community Health (11/13), Smoking Cessation (8/13), STDs/Family Planning (6/13), Diabetes Prevention, Harm Reduction and Immunization (5/13). The Nutrition program did not have any participants identify it as one of the most utilized prior to Healthy at Home.

#### Challenges During COVID-19

Sixty-nine percent (9/13) of the LHDs in this study reported that their programs were impacted following the Healthy at Home order. When asked about barriers faced throughout the order’s enforcement period, all respondents (13/13) identified limited internet access as the most common barrier for clients. The second-most reported barriers to LHD provision and client access of essential services were lack of transportation (69%; 9/13), lack of cellular access (69%; 9/13), and lack of satellite health locations (69%; 9/13). Additionally, LHD respondents were asked whether challenges with funding or supply-chain access affected their operations during the COVID-19 pandemic. Being able to find individuals via cell phone was a common challenge affecting 92% (12/13) of LHDs, with one LHD respondent stating, “So many folks use cell pre-paid phones and run out of minutes and then end up with different numbers” and “There is a lack of internet accessibility and cell phone reception which can make completing the 30-minute visits difficult.” Having adequate personnel was also identified as a common challenge by 77% of LHDs (10/13). Only one LHD reported facing challenges with electronic hardware, such as laptops and webcams, or with outdated software.

#### Success of Telehealth Implementation

When asked about how utilizing telehealth affected participation rates for frequently used programs, 54% (7/13) of LHDs reported a *substantial increase* in participation rates for WIC and HANDs, compared with the same period in 2019. For example, one LHD respondent stated, “WIC currently has the highest participation rates we have ever seen, with a 98.21% participation rate. This success has been partly due to the waivers the USDA provided for the Commonwealth, which allowed WIC certifications to be done remotely. The WIC program also received nearly $60,000 to purchase tablet computers to help the clerks register remotely.”^21^ The remaining 46% (6/13) reported a “moderate increase” in participation rates for WIC and HANDs.

Those who reported a *substantial increase* in participation had started using telehealth before Healthy at Home, while those with “moderate increase” in participation started offering telehealth during Healthy at Home, with one LHD respondent stating, “WIC had already been successful before Healthy at Home.” According to personal communication with three LHD respondents, participation rates for WIC increased as follows: 31% increase from February to November 2020 (Pike County Health Department); 10% increase from April 2020 to December 2021 (Floyd County Health Department); 8% increase from January 2019 to December 2020 (Kentucky River Health Department). Additionally, respondents reported that the main contributor to the success of telehealth was the ease of use for clients, as it was described as “user-friendly” and more convenient to access from home than face-to-face care. Telehealth did not require travel nor risk of exposure to COVID-19. One LHD participant stated, “WIC is education-based, so it was easier to transition [to telehealth].” LHD reported not utilizing telehealth for those essential services that often require in-person interaction, such as immunization programs, STDs/Family Planning, and Harm Reduction. All respondents noted that telehealth was not implemented for these programs either because clients would not feel comfortable using a virtual platform or because providing the service via telehealth was not possible.

### Impact of COVID-19 on Public Health Communication

Respondents were asked to describe what forms of communication were commonly used to connect with clients during the Healthy at Home order. All participants indicated they preferred using social media or radio to communicate with their local population; the second-most preferred means of communication were flyers (92%; 12/13) and websites (85%; 11/13).

When asked about the most frequently used media sources for health awareness and education, LHD respondents reported they preferred using social media (100%; 13/13), radio or TV (92%; 12/13), and the newspaper (92%; 12/13). Similarly, all LHD respondents reported having to increase the use of these media platforms during the COVID-19 pandemic to provide updates on preventive measures, such as proper mask use, social distancing, district testing locations, isolation, and quarantine. Furthermore, all participants reported using external research data from CDC as the primary resource for information content and reliability.

#### Social Media Online Health Communication

From November 1, 2019, to December 31, 2020, a total of 2,135 LHD Facebook posts related to COVID-19 health education were identified for five LHDs participating in the study. The Kentucky River District Health Department (covering seven counties) accounted for the highest proportion of posts at 36% (777/2,135), followed by the Floyd County Health Department at 19% (412/2,135), and the Pike County Health Department at 19% (415/2,135). The highest percentage of Facebook posts occurred in April (14%; 301/2,135) and July (10%; 223/2,135). The Kentucky River District Health Department received an average of 13 feedback responses per post (1,585 responses to a total of 122 posts) versus 40 feedback responses per post (2,909 responses to a total of 73 posts) for Pike County Health Department in April; 26 feedback responses per post (1,748 responses to a total of 67 posts) versus 74 feedback responses per post (4,392 responses to a total of 74 posts), respectively, in July.

Of the 2,135 COVID-19-related LHD Facebook posts created during November 2019–December 2020, 68,284 feedback responses were submitted by the public. Of the 68,284 feedback responses, 99.70% (68,082/68,284) were positive, 0.23% (155/68,284) were negative, and 0.07% (47/68,284) were neutral or inconclusive. Floyd County Health Department had the highest proportion of responses at 39% (26,380/68,284), followed by Pike County at 25% (16,733/68,284), and the Kentucky River Health Department at 18% (12,325/68,284). The highest number of all feedback responses were observed in April and July at 14% (9,469/68,284) and 16% (10,832/68,284), respectively.

## DISCUSSION

The COVID-19 pandemic posed new challenges to LHDs serving counties in Appalachian Kentucky that further impacted access to health care for rural communities already challenged by economic and health inequities prior to the pandemic. Appalachian counties have some of the highest rates of social vulnerability in the country, and the counties that participated in this study are some of the most vulnerable.^6^,^22^ Although all 13 counties in this study identified limited access to cellular devices and internet broadband as the primary challenge during the Healthy at Home order’s effective period, there were additional hurdles faced by LHDs. Small LHDs—serving populations of 10,000 or fewer—faced more challenges with clients’ ability to access transportation and with the availability of resources or supplies (e.g., food and water, personnel, and personal protective equipment) compared with larger LHDs. Public health has long been underfunded and is disproportionately lower in rural areas than urban counterparts, compounding the challenges to ongoing LHD operations within Appalachia.^6^,^23^ On average, small population counties had the highest rates of diabetes and obesity, the highest rates of COVID-19 cases per 100,000 population, and the greatest numbers of COVID-19-related deaths, which is consistent with impacts of the pandemic nationally.^20^ The rapid spread of COVID-19 resulted in a shift in how LHDs provide essential public health services towards online health options, particularly for programs with less need for in-person visits or follow-ups. Our findings show that among the LHDs assessed, those that transitioned to telehealth *before* the Healthy at Home order experienced an increase in participation for WIC and HANDs programs compared to those that transitioned after the order. The increased participation in WIC and HANDs for these relatively small counties, compared with large counties, demonstrated that when telehealth options are available, people will seek services that otherwise might not have been accessible due to transportation or other health equity issues.

These findings help illuminate the impact of COVID-19 on frequently used programs in Appalachian Kentucky and highlight how LHDs implemented strategies to continue operations. The LHD social media posts and public feedback responses from November 1, 2019, to December 31, 2020, reached a wider audience than usual and were a major source of information in these communities. Results from feedback responses and social media posts suggest a large increase in the public interest for all the LHDs during the early phase of the COVID-19 pandemic, particularly in April and July 2020. This coincides with a chronological sequence of events, with the first confirmed case of COVID-19 in March and the Healthy at Home order in July. This study’s findings appear to demonstrate that increases in the number of Facebook posts led to an increase in the number of feedback responses from the public, with most responses being positive.

While this study provides insights into the impact of COVID-19 on essential public services and into online health communication provided by LHDs, the results are subject to several limitations. County demographics were limited to only select health data and population size due to limits on publicly available data. The self-reported information from LHD directors (or their representatives) who agreed to participate may be influenced by both selection and response bias. Not all respondents were able to be interviewed and required follow up later by email, which could have resulted in recall bias. Using qualitative data from social media posts and feedback responses does not reflect all sources of communication or public opinion, and these data may not be representative of all populations in the LHD communities. Finally, these results are limited to the LHD that participated and are not generalizable to all 54 counties in the ADDs of Appalachian Kentucky. Future studies should aim to understand where community members get health information most frequently, the impact of telehealth on the quality of and access to care, and how essential public health services were impacted over time for a wider range of rural Appalachian counties.

Telehealth appears to have been an effective option to provide essential public health services in three ADDs in Eastern Kentucky. Encouraging greater use of telehealth, expanding options for participation, adapting laws governing telehealth, furthering options for reimbursement, and increasing access to online health communication may improve health services for rural populations in Appalachian Kentucky.^5^ The continued expansion of high-speed internet throughout the region is essential for improving health outcomes for underserved rural populations. Through the efforts championed by SOAR, the success demonstrated by maintaining essential public health services during the pandemic, and the greater flexibility with federally funded services, LHD in Appalachian Kentucky can more effectively serve their populations.

**SUMMARY BOX**

**What is already known about this topic?**

Appalachian Kentucky has higher rates of health disparities and challenges with access to care made worse during the COVID-19 pandemic stay-at-home orders.

**What is added by this report?**

Telehealth appears to have been an effective option to provide essential public health services in three districts in Eastern Kentucky.

**What are the implications for future research?**

Encouraging greater use of telehealth and increasing access to online health communication may improve health services for rural populations.

## Supplementary Information



## Figures and Tables

**Table 1:** Population Demographics and Health Outcomes by Area Development District (ADD), 2019

ADD Location	Population	Obesity prevalence	Diabetes prevalence	Poverty prevalence	No. drug overdose deaths*;†
**Cumberland Valley** §	238,531	43%	20%	27%	76
Rockcastle County	16,820	43%	19%	21%	^**^
Jackson County	13,369	43%	22%	28%	^**^
Clay County	20,368	42%	18%	33%	8
**Kentucky River**	105,992	45%	18%	31%	60
Knott County	15,260	44%	22%	31%	7
Lee County	6,881	43%	21%	35%	8
Leslie County	10,283	48%	16%	32%	^**^
Letcher County	22,295	44%	23%	29%	7
Owsley County	4,442	43%	18%	36%	^**^
Perry County	26,624	48%	15%	24%	5
Wolfe County	7,207	43%	18%	30%	^**^
Breathitt County	13,000	43%	20%	29%	6
**Big Sandy** ¶	142,726	44%	24%	24%	61
Floyd County	36,456	44%	23%	27%	13
Pike County	59,497	43%	24%	20%	23

NOTES:

* Confirmed drug overdose deaths from any substance are defined as any death listing one of the following ICD-10 codes in the “underlying cause of death” field of the death certificate: X40–X44, X60–X64, X85, Y10–Y14.

† The Kentucky Office of Vital Statistics does not report an exact number if county deaths are less than five persons and does not calculate per capita for less than twenty.^15^

§ Total population for Cumberland Valley ADD includes population from eight counties, including Bell, Harlan, Knox, Laurel, and Whitley.

¶ Total population for Big Sandy ADD includes population from five counties, including Johnson, Magoffin, and Martin.

**Table 2:** COVID-19 Summary Report by Area Development District (ADD) and County in 2020

ADD Location	Total cases	Total deaths	Incidence rate per 100K	Total cases per 100K	Fatality rate
**Cumberland Valley ADD** *	15,059	116	**69**	**6,891**	0.8%
Rockcastle County	867	8	42	5,155	0.9%
Jackson County	858	20	46	**6,418**	2.3%
Clay County	1,655	8	**129**	**8,125**	0.5%
**Kentucky River ADD**	6,129	69	49	5,783	1.1%
Breathitt County	619	3	**74**	4,762	0.5%
Knott County	741	14	29	4,856	1.9%
Lee County	1,046	18	31	**15,201**	1.7%
Leslie County	475	1	38	4,619	0.2%
Letcher County	1,061	4	**72**	4,759	0.4%
Owsley County	314	10	**78**	**7,069**	3.2%
Perry County	1,574	16	**51**	5,912	1.0%
Wolfe County	299	3	24	4,149	1.0%
**Big Sandy ADD** †	7,331	76	37	5,136	1.0%
Floyd County	1,844	16	**50**	5,058	0.9%
Pike County	2,989	33	**67**	5,024	1.1%

Notes:

Numbers are shown in **bold** if higher than the Commonwealth’s average.

* Total data for Cumberland Valley ADD includes total data from eight counties, including Bell, Harlan, Knox, Laurel and Whitley.

† Total data for Big Sandy ADD includes total data from five counties, including Johnson, Magoffin and Martin.
