# Supplementary material for: COVID-19 Pandemic Impact on Essential Public Health Services and Online Health Communication—Appalachian Kentucky, March–December 2020
Source: J Appalach Health. 2022 Jul 1;4(2):8–25. doi: 10.13023/jah.0402.03 (PMC10629872; doi:10.13023/jah.0402.03)
Supplement: Supplementary file 1 [file 4.2.3_Riggs_AdditionalFiles.pdf]

Appendix A. Kentucky Local Health Department Essential Services

Health Access Nurturing Development Services (HANDS)

Goal: to support a home visiting program for pregnant moms-to-be and new parents that support building healthy, safe environment for the optimal growth and development of children.

http://www.kyhands.com/about/

Special Supplemental Nutrition Program for Women, Infants, & Children (WIC)

Goal: to support a free, voluntary program that supports new parents by providing expertise and tools in all aspects of parenting.

https://chfs.ky.gov/agencies/dph/dmch/Pages/default.aspx

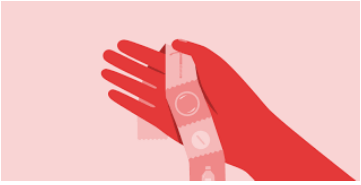

STDs/Family Planning

Goal: to educate and counsel individuals about birth control methods and protection from sexually transmitted diseases (STDs).

https://chfs.ky.gov/agencies/dph/dwh/Pages/familyplanning.aspx

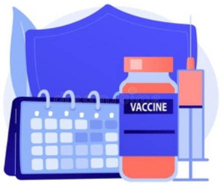

Immunization Program

Goal: to prevent and reduce the risk and incidence of vaccine preventable diseases. The Vaccines for Children Program was created to ensure all children can access life-saving vaccines.

https://chfs.ky.gov/agencies/dph/dehp/Pages/vfc.aspx

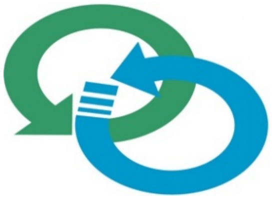

Harm Reduction

Goal: to provide services that reduce the harms associated with substance abuse. Needle exchange programs provide sterile needles and syringes and safe needle and syringe disposal at no cost.

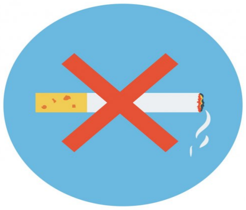

Tobacco Prevention and Cessation

Goal: to reduce the number of preventable deaths due to tobacco by encouraging youth not to smoke and providing resources to quit smoking.

https://chfs.ky.gov/agencies/dph/dpqi/cdpb/Pages/tobcessation.aspx

https://chfs.ky.gov/agencies/dph/dehp/hab/Pages/kyseps.aspx

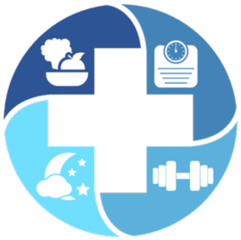

Diabetes Prevention

Goal: to support a coalition of public and private partners that encourage people at risk for type-2 diabetes to participate in evidence-based lifestyle change programs to reduce their risk of the disease.
https://chfs.ky.gov/agencies/dph/dpqi/cdpb/Pages/diabetes.aspx

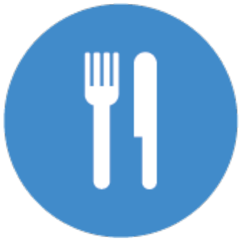

Nutrition

Goal: to prevent obesity by supporting policy and environmental strategies to make healthy eating and active living accessible and affordable.
https://chfs.ky.gov/agencies/dph/dmch/nsb/Pages/default.aspx

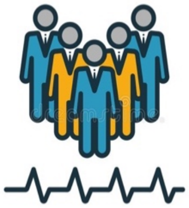

Community Health

Goal: to promote health and prevent disease through technical assistance and continuing education
https://chfs.ky.gov/agencies/dph/dpqi/cdpb/Pages/chwp.aspx

Appendix B: Survey Template

Local Health Department Questionnaire for (insert county name/district)

Section A

1. Describe your population's demographics, particularly those demographics relating to a health outcome?

Population	Obesity rate	Diabetes rate	Poverty	Overdose fatalities

Section B

1. Prior to healthy-at-home, what services and programs did your health department offer and which were most utilized by your population?

Program	Utilized or not utilized	Other
WIC		
HANDS		
STDs /Family Planning		
Immunizations		
Harm Reduction		
Tobacco Prevention and Cessation		
Diabetes Prevention		
Nutrition		
Community Health		

2. Prior to healthy-at-home, did you utilize telehealth to provide care? Do you plan to continue offering virtual services after fully reopening? This will depend on the state and federal approval to do so for many of the programs that we offer.

Program	Telehealth before	Telehealth during healthy-at-home	Telehealth after full reopening	Other

3. After healthy-at-home, has it been more difficult to reach certain areas of the population?

	Transportation for home visits or	Peoples without Internet access	Peoples without cellular access	Satellite health locations

	clients having to stay at home			(school nurse, alternate off-site clinics)
Yes				
No				
Why				

4. After healthy-at-home, were there challenges with funding or supply chains to access necessary resources that affected your operations?

	PPE	Food/ water	Family planning/toil etries	Narcan	Internet	Cellular	Pe rs on ne l	Electron ic Hardwa re (webca ms, laptops, tablets, etc.)	Grants or contra cts
Yes									
No									
Why									

5. How has healthy-at-home affected the programs or services you offer?

6. Have telehealth or virtual services been factors in whether a program was successful during healthy-at-home?

7. Were there specific aspects of these programs that helped or hindered an effective transition?

8. What are your plans to respond to a resurgence of COVID-19?
    a. Such as:
    b. Addressing gaps in supply chain access
    c. Addressing gaps in access to communities
    d. Addressing gaps in telehealth
        i. Federal/state regulations
    e. Mass immunization plans-
    f. PPE distribution-
    g. Public health messaging and outreach
    h. Community leadership task forces-

Appendix C Public Health Communications During COVID-19

Section C

1. What means of communication did you utilize to connect with your population during the Healthy-at-Home order?

Form of Communication	Used? (Y/N)	Frequency? (One post/article/video upload per day? Two times a day?)	Comments
Social Media			
Websites			
Radio			
TV (WYMT)			
Newspaper			
Press Conferences			
Flyers			
Bulk Mailing			
Other			

2. How do you receive feedback *(social media, letters, calls, etc.)*?

3. What feedback do you receive from the community?

4. What kind of content pertaining to COVID-19 did your public health messaging contain?

5. What sources did your research/information stem from *(internal, external)*?

6. Are there concerns in your ability to reach clients who lack telecommunications?

7. Which methods of communication are most effective for your population?

8. What forms of media did you use for health promotion/education? *(Please check the appropriate box/es.)*

☐ Social Media
☐ Websites
☐ Radio
☐ TV (WYMT)
☐ Newspaper
☐ Activity Packets
☐ Curbside service materials
☐ Press conferences
☐ Other (Please explain):
